# Difficult Terrains of Research Publications Faced by Researchers: A Cross-Sectional Study

**DOI:** 10.7759/cureus.64787

**Published:** 2024-07-18

**Authors:** Ravindran Chirukandath, Gopika Sunil, Vipin Balakrishnan, R Ashitha Menon, Soorya Gayathry P, Sumin V Sulaiman, Marius George, Keerthana Mohan, Dona Maria Joseph

**Affiliations:** 1 General Surgery, Government Medical College Thrissur, Thrissur, IND; 2 Dermatology, Government Medical College Thrissur, Thrissur, IND; 3 Obstetrics and Gynaecology, Amala Institute of Medical Sciences, Thrissur, IND; 4 Physiology, Government Medical College Thrissur, Thrissur, IND

**Keywords:** academics, journals, medical education, publications, research

## Abstract

Background

The publication of scholarly work in peer-reviewed journals is a well-established method for disseminating knowledge and findings to a global audience. However, the publishing process is constantly evolving and encountering various obstacles that hinder progress. Despite a significant increase in the number of research projects undertaken, there are few studies evaluating the challenges faced by investigators in publishing their research. This study aims to identify the factors and elements that influence the publication process after the completion of research.

Methods

This study included 759 projects approved by the Institutional Ethics Committee (IEC) from 2016 to 2021 at a tertiary care centre in South India. A list of these approved projects was analysed for overall output in terms of publication and completion. Investigators were contacted and interviewed using a validated, 15-question survey to identify various factors influencing scientific publications.

Results

A total of 759 projects approved by the IEC from 2016 to 2021 were analyzed. It was found that only 36.72% of studies were completed by faculty members, and the publication conversion rate was 34.24%. A single-point analysis showed a statistically significant lower conversion rate for resident articles (p = 0.032). The 15-point analysis detailed the factors influencing publication conversion, revealing that the majority of researchers publish based on academic and research interests (68.89% and 72.12%, respectively). Various deterrents to publication, such as study design, statistical analysis, journal selection, and knowledge about journal submission, were identified. Notably, 98.4% of researchers expressed a desire to publish more in the future, highlighting the importance of this study.

Conclusion

The study highlights areas that require attention to facilitate and augment research. It identifies the real gaps in the publication process and suggests points of intervention needed to enhance the research environment, increase publication rates, and establish demand-based research support units in the medical education sector.

## Introduction

The publication process in India is still evolving, with multiple barriers impeding the successful dissemination of research findings. Despite a significant increase in the number of research projects undertaken, the rate of resulting publications remains limited. There is a scarcity of studies evaluating the challenges faced by investigators in publishing their research [1,2]. Numerous factors influence the quality of scientific publications, including rigorous methodology, valid results, significance of findings, journal reputation, peer review process, funding sources, collaboration, timeliness, and writing quality [3,4].

A detailed analysis of the difficulties encountered by investigators in publishing their research and the factors influencing the publication output of approved research projects has not been thoroughly explored in India, particularly in Kerala. This study aims to identify the possible factors and elements that influence the publication process following the completion of research. It focuses on the challenges and factors affecting the publication of research findings from projects approved by the Institutional Ethical Committee (IEC) at Government Medical College in Thrissur, Kerala, India.

The study seeks to address the obstacles faced by investigators in publishing their research and identify the factors influencing the publication output of approved research projects. It includes a comprehensive review of the challenges encountered during project execution and publication, the study setting, research questions, and the methodology employed. The study examines the role of institutional support, availability of resources, and training in research methodology as potential barriers to successful publication. It also investigates the influence of external factors such as journal selection criteria, peer review processes, and publication costs. By identifying these factors, the study aims to propose strategies to enhance the research environment and improve the publication rate of approved projects. Additionally, the study will provide recommendations for policy changes at institutional and governmental levels to support researchers in overcoming these barriers. The ultimate goal is to foster a culture of research and publication, thereby contributing to the advancement of medical science and improving healthcare outcomes in the region.

## Materials and methods

We conducted a cross-sectional study of all projects approved by the IEC over six years, spanning from January 2016 to December 2021, at Government Medical College in Thrissur, a tertiary-level institution in central Kerala, India. The IEC of Government Medical College Thrissur issued approval to conduct the study (approval number: IEC/GMCTSR/234/2021). A total of 759 projects were analyzed. The primary objective was to quantitatively assess the publication status of these approved research projects and identify the factors influencing researchers in the scientific publication of their work.

A comprehensive list of IEC-approved projects from the specified period was examined for overall output in terms of publication and completion. We compiled all studies approved by the IEC, and the principal investigators (PIs) were contacted. Data were collected through direct or telephonic interviews using a validated 15-item questionnaire designed to identify various factors influencing scientific publications. The inclusion criteria encompassed all 759 projects approved between January 1, 2016, and December 31, 2021. We included PIs who could be contacted directly or via telephone. Exclusion criteria consisted of PIs who could not be reached or were unwilling to participate in the study.

Data collection was conducted using a standardised proforma. The data were then collated and analysed using Epi Info software (Centers for Disease Control and Prevention, Atlanta, GA). A master chart was created to facilitate detailed analysis and presentation of the results.

## Results

**Figure 1:**
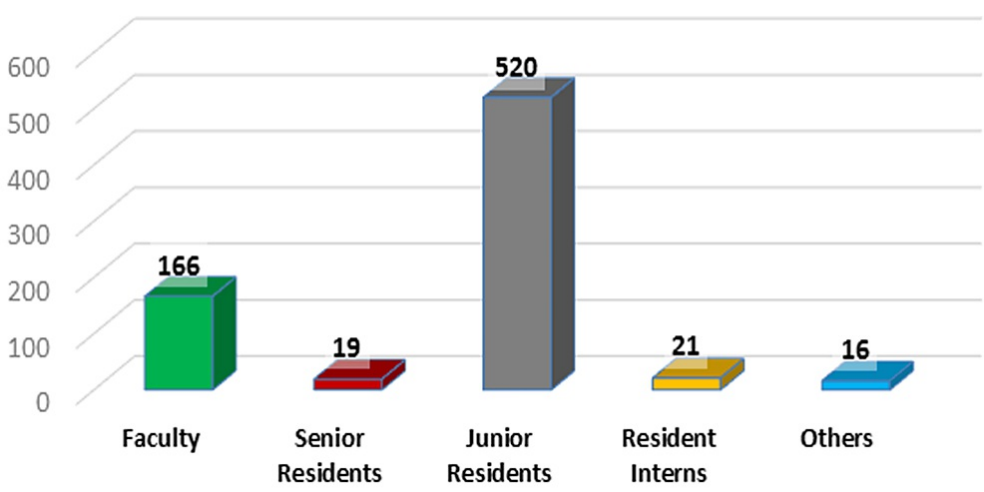
A break-up of the total projects approved by the Institutional Ethics Committee

In this interview-based study, we incorporated a total of 759 studies approved by the IEC between 2016 and 2021 (Figure 1). However, data were successfully obtained from only 321 principal investigators. The majority of the approved studies were conducted by junior residents, comprising nearly 69% of the total, followed by faculty members. Notably, medical students and resident interns also demonstrated significant interest in participating in research activities.

**Figure 2:**
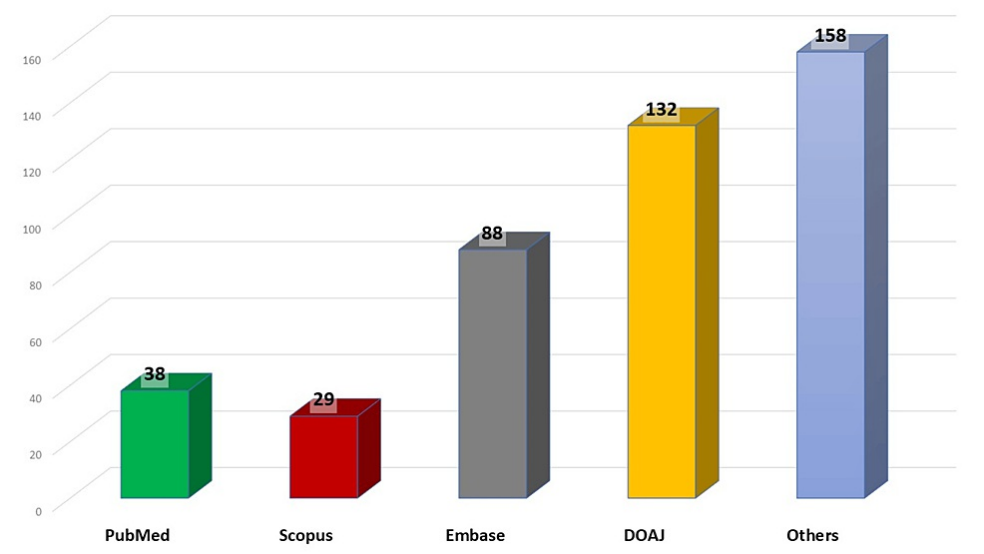
Number of publications in various databases Embase: Excerpta Medica database; DOAJ: Directory of Open Access Journals

Among the 321 principal investigators interviewed it was found that only 189 studies were published. Of these, approximately 62% were published in national journals, with very few appearing in international databases such as PubMed and Scopus (Figure 2). Faculty studies accounted for only 36.72% of the total approved studies, with a conversion rate of 34.24%.

The resident group demonstrated a statistically significant lower rate of converting studies into publications (p = 0.0032). The majority of researchers published based on academic and research interests, with 68.89% and 72.12%, respectively. When asked about their desire to publish, 98.4% of investigators expressed a wish to do so.

**Figure 3:**
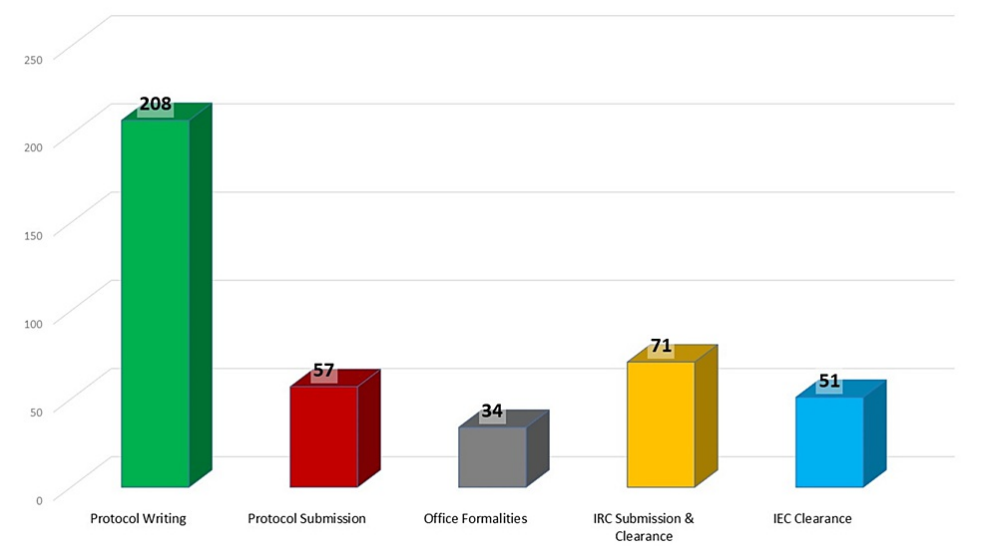
Difficulties faced during project execution IRC: Institutional Research Committee; IEC: Institutional Ethics Committee

The difficulties in publishing research were analysed under two categories: project execution and project publication. During project execution, the most challenging aspect was protocol writing and submission, accounting for nearly 62% of the problems faced by investigators, followed by Institutional Research Committee (IRC) clearance. Other issues included completing office formalities and obtaining IEC clearance (Figure 3).

**Figure 4:**
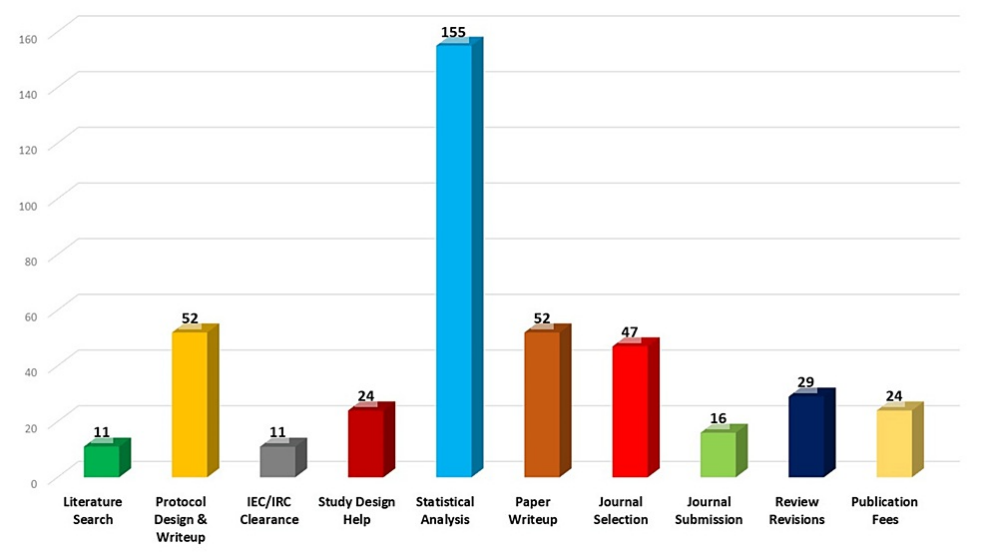
Difficulties faced in project publication

The major hindrance to paper publication was performing a proper statistical analysis. Other significant factors included paper writing and journal selection. Investigators noted that even if they could write a good paper with accurate statistical analysis, selecting an appropriate journal and following up was difficult (Figure 4).

**Figure 5:**
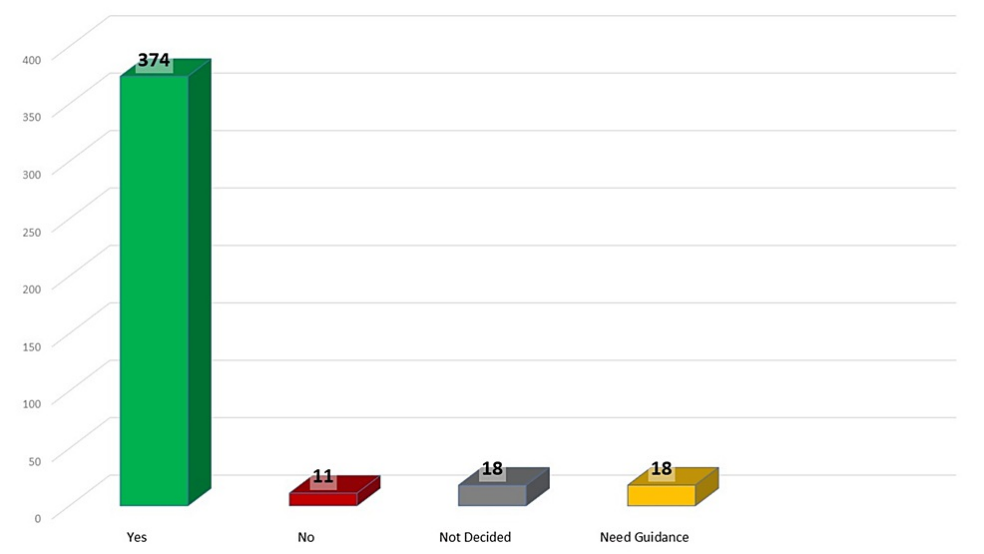
The answers to the question "Do researchers want to publish?"

The majority of investigators expressed a desire to publish again, indicating the need for mechanisms to improve the research environment, motivation, and assistance (Figure 5). Additionally, they said needed to select topics oriented towards publication.

## Discussion

This study analysed the publication status of 759 research projects approved by the IEC between 2016 and 2021. The findings reveal that only 36.72% of the approved studies were completed, with a publication conversion rate of 34.24%. Additionally, the majority of researchers published based on academic and research interests, with 98.4% expressing a desire to publish more in the future.

The significance of this study lies in its identification of the factors influencing the publication of research findings from approved projects, providing valuable insights into the challenges faced by investigators [5]. By quantitatively analysing the publication status and conducting interviews, the study not only identified barriers but also highlighted the strong inclination of researchers towards future publications [6].

Various reasons contribute to the non-publication of research findings, including ongoing manuscript preparation, studies still in progress, and biases due to negative results, which are clearly unacceptable [7]. Other documented reasons include journal rejections (6%), fear of non-acceptance (12%), lack of motivation, and low prioritisation of publication [8, 9]. The difficulties encountered by investigators during project execution and publication were substantial. The major hindrance to paper publication was performing proper statistical analysis. Additionally, challenges in project execution included protocol writing and submission, which accounted for almost 62% of the problems faced by investigators. Other issues included obtaining Institutional Research Committee clearance, completing office formalities, and IEC clearance. Further, difficulties in the paper write-up and journal selection were significant obstacles in the publication process.

The analysis of publication output revealed that the majority of publications were in Indian journals and those indexed in The Directory of Open Access Journals (DOAJ), the Excerpta Medica database (EMBASE), Crossref, and other databases. Less than 6.2% of the articles were published in PubMed-indexed journals, likely due to several factors [10]. Time constraints lead researchers to focus on journals with high impact factors, which typically accept articles with positive results [11]. Additionally, poor study designs, repetitive research, and duplication of study results contribute to non-publication in high-impact, PubMed-indexed journals [12].

The Bachelor of Medicine, Bachelor of Surgery (MBBS) curriculum is the primordial point where the fundamentals and outline of research should be introduced. By integrating research education early in the curriculum, we can encourage more students to take advantage of publication opportunities, such as those offered by The Indian Council of Medical Research (ICMR). This early exposure not only promotes a research-oriented mindset but also equips students with the necessary skills to contribute meaningfully to medical science. The study identifies points of intervention needed to enhance the research environment, improve publication rates, and establish demand-based facilitatory research units in the medical education sector [13,14]. Addressing the challenges and barriers faced by investigators during project execution, such as protocol writing, submission, and IRC clearance, as well as obstacles in project publication, including statistical analysis, paper write-up, and journal selection, is crucial.

Furthermore, the study highlights the need for onsite assistance and the desire of most researchers to publish more in the future. These findings underscore the necessity for interventions aimed at improving the publication conversion rate, addressing specific challenges faced by different groups, and fostering a more supportive research environment within the medical education sector [15].

## Conclusions

The publication process is still evolving in this country, and multiple barriers impede the process of research publication, including time constraints. The number of research projects being undertaken has increased drastically, but publications are limited. By quantitatively analysing the publication status and conducting interviews, the study not only identified the barriers but also revealed the researchers' strong inclination towards future publications. Ultimately, the findings offer a compelling basis for devising interventions aimed at improving the publication conversion rate, addressing specific challenges faced by different groups, and fostering a more facilitatory research environment within the medical education sector. 
